# Preparation of a Novel Type of Zwitterionic Polymer and the Antifouling PDMS Coating

**DOI:** 10.3390/biomimetics7020050

**Published:** 2022-04-21

**Authors:** Xutao Ma, Xiaohui Fu, Jing Sun

**Affiliations:** 1 College of Polymer Science and Engineering, Qingdao University of Science and Technology, Qingdao 266042, China; 15552266138@163.com (X.M.); fuxiaohui@iccas.ac.cn (X.F.); 2 State Key Laboratory of Supramolecular Structure and Materials, College of Chemistry, Jilin University, Changchun 130012, China

**Keywords:** zwitterionic polymers, antifouling coatings, ring-opening polymerization

## Abstract

As awareness of environmental protection increases, environmentally friendly coatings have been receiving great interest. Zwitterionic polymers are considered promising candidates due to their biocompatibility and excellent antifouling properties. In this paper, a type of polypeptoid containing zwitterions on the side chain was synthesized via ring-opening polymerization (ROP) and post-modification. This obtained polypeptoid was subsequently grafted onto the surface of polydimethylsiloxane (PDMS) via plasma and UV-induced surface polymerization. Surface morphology and protein adsorption tests of the resulting coating were systematically carried out. The results show that the modified coating has excellent antifouling properties and thus has great potential for environmentally friendly coating applications.

## 1. Introduction

Biofouling is the microbial precipitation or formation of a biofilm after a surface of a material adsorbs microorganisms, and it is the main cause of medical devices becoming infected [1,2,3]. For medical-related applications, there is an urgent need to develop surface coatings that prevent such infections. However, the rapid development of medical devices often comes with challenges [4,5,6,7]. For example, implants are often recognized as “foreign objects” by the host’s body [8]. The immune system reacts based on complex signals that lead to complications around newly implanted materials [8]. This process is known as the foreign body response (FBR); it is a natural protective mechanism of the host that greatly affects the function of implanted materials. In this context, developing a type of biological coating with antifouling properties is a matter of utmost importance. Coatings modified with hydrophilic compounds can form a hydrated layer after binding to water molecules within the host’s body, which can inhibit the adhesion of organic substances [9]. Common hydrophilic polymers include polyethylene glycol and zwitterionic polymers. Poly (ethylene glycol) (PEG) and its derivatives exhibit antifouling effects on various proteins, but their auto-oxidation properties limit their application in many scenarios [10,11,12,13]. Zwitterionic polymers remain electrically neutral overall and are highly hydrophilic in an aqueous solution. They can not only prevent dead bacteria from adhering to the surface of the material but also have excellent antifouling properties [14,15,16,17,18]. Zwitterionic polymers, such as phosphobetaines, poly (sulfobetaine) [19,20], and poly (carboxybetaines) [21], have been recognized as emerging antifouling materials. Due to strong electrostatically induced hydration, zwitterionic polymers form tightly bound layers of water around their polymer chains, which can repel nonspecific proteins and prevent them from being adsorbed. Due to their strong anti-adsorption properties and biocompatibility, zwitterionic polymers hold great potential in the field of biomaterials.

Recently, a new strategy to prepare polymer brushes by UV light-induced surface-initiated atom transfer radical polymerization (SI-ATRP) has received great attention [22]. This photopolymerization method has many advantages such as facile and efficient procedures and avoidance of toxic catalysts [23]. SI-ATRP technology is well established to construct polymer brushes with flexible molecular structures, high graft density, and long-term surface sustainability [24,25,26]. Polydimethylsiloxane (PDMS) is a biocompatible material that is widely used for biomedical research and technology. Polypeptoids, also known as nitrogen-substituted polyglycines, are one of the most important peptidomimetic polymers, and they hold great potential in various biomedical applications [27,28]. With a similar backbone structure to polypeptides, polypeptoids exhibit great biocompatibility and bioactivity, which are excellent properties for biomedical applications [29,30,31]. In recent years, ring-opening polymerization (ROP) has been used to synthesize polypeptoids with narrow molecular weight dispersions and high molecular weights [32,33,34]. In this study, we have developed a facile method for carrying out surface grafting in the aqueous phase with plasma and UV lamps. First, we employed benzylamine (Bn-NH_2_) to initiate the ROP of N-allyl N-carboxyanhydride (NAG-NCA), and then we combined click chemistry and post-modification to synthesize polypeptoids with zwitterions on their side chains. Exposure to an argon plasma generated activated peroxide or hydroperoxide groups as surface initiators, which then allowed the zwitterionic polymer to be grafted onto the surface of the PDMS under UV irradiation. The as-prepared coating had excellent compatibility with neglected non-specific protein adsorption, meaning it holds great potential for application in future biomedical materials and devices.

## 2. Materials and Methods

### 2.1. Materials and Instruments

Triethylene glycol monomethyl ether and epichlorohydrin were purchased from Aladdin reagent. Benzylamine (98%) was purchased from Shandong West Asia Chemical Co., Ltd., Shandong, China, Dichloromethane (DCM), tetrahydrofuran (THF), and hexane were purified by activated alumina columns. Triethylamine (TEA) and phosphorus trichloride (PCl_3_), Shanghai San Chemical Technology Co., Ltd. All other chemicals were purchased from commercial suppliers and were used without further purification unless otherwise noted.

The ^1^H NMR spectra were recorded on a Bruker AV400 FT-NMR spectrometer. Tandem gel permeation chromatography (GPC) was conducted using an SSI connected to a Wyatt Optilab DSP at a flow rate of 1.0 mL/min in DMF, using 0.02 M LiBr as the eluent at a temperature of 50 °C. The concentration of all samples was approximately 5–10 mg mL^−1^. Calibration was performed using polystyrene (PS) standards. The mercury high pressure UV lamp (250 W, GGZ250, Shanghai Jiguang Special Lighting Electrical Factory, Shanghai, China) was used for irradiation of peptoid solution during mercaptopropionic acid-ene click chemistry. Fourier transform infrared (FTIR) were recorded within a range of 2000 to 1400 cm^−1^ on a JASCOFT/IR-4700 spectrofluorometer. Prior to any measurements being taken, all sample solutions were dropped on KBr plates. Confocal laser scanning microscopy (CLSM) images were taken with a Nikon C2 plus (Nikon Corporation, Tokyo, Japan). The AFM studies were performed using tapping mode AFM under ambient air. The fabricated coating samples were placed on freshly cut mica and then subjected to AFM testing. Minimal processing of the images was conducted using Nanoscope Analysis software from Bruker.

### 2.2. Synthesis of Epoxide Terminated Oligo (Ethylene Glycol)

The epoxide terminated oligo (ethylene glycol)s were synthesized according to the reported literature method [34]. Triethylene glycol monomethyl ether (18 g, 116.8 mmol) was first dissolved in water as the solvent, and an aqueous solution of NaOH (30.6 g, 379.1 mmol) and tetrabutylamine hydroxide (50% aqueous solution water) (14.2 mL, 24.8 mmol) was then added within 5 min. Epichlorohydrin (30 mL, 365.4 mmol) was added with a constant pressure dropping funnel under ice bath conditions over 3 min. The mixture was reacted in a round-bottom flask for 24 h at 37 °C. Deionized water was added, and the mixture was extracted three times with ethyl acetate. The organic layer was washed three times with saturated brine and dried over anhydrous magnesium sulfate. The solvent was removed using a rotary evaporator to give a colorless liquid with a yield of 71.51%.

### 2.3. Polymerzation of N-Allyl N-Carboxyanhydride (PNAG)

The *N*-allyl *N*-carboxyanhydride (NAG-NCA) was synthesized according to a previous method [33]. NAG-NCA (1.7 g, 0.13 mol) was dissolved in THF (100 mg/mL) under a nitrogen atmosphere. The solution of benzylamine in anhydrous THF (7.86 wt%) was then added to the polymerization tube. The mixed solution was then reacted at 55 °C. The polymerization reaction was detected by FTIR. When the characteristic peaks of NAG-NCA disappeared completely, the mixed solution was precipitated with ether in an ice bath, which was repeated three times. The resulting white solid was collected by centrifugation (71.8% yield).

### 2.4. General Procedure for Modification of PNAG with 3-Mercaptopropionic Acid (PNAG-COOH)

PNAG was dissolved (100 mg/mL) in *N*,*N*-dimethylformamide in a Shrek bottle, followed by the addition of benzoin dimethyl ether (DMPA). Under a nitrogen atmosphere, mercaptopropionic acid (5.97 mmol, 0.45 mL) was rapidly added. After returning the solution to room temperature, it was continuously irradiated under 400 nm UV light for 4 h, and then the solution was precipitated with ice ether to obtain a yellow oily liquid (0.13 g, 73.9% yield).

### 2.5. General Procedure for the Post-Modification of PNAG-COOH

The PNAG-COOH was dissolved in formic acid, and then epoxides (OEG_3_: allyl glycidyl ether = 10:1) were added under a nitrogen atmosphere. The reaction was stirred vigorously at 37 °C for 60 h and then precipitated and purified in an excess amount of ice ether (~70% yield).

### 2.6. Preparation of Zwitterionic Polymer Coatings

The PDMS silicone rubber slides were prepared following the product instruction manual. In brief, the curing agent and monomer were mixed in a mass ratio of 1:10 and cured in a glass dish at 60 °C for 16 h. The cured silica slides were first rinsed with ethanol, soaked in hexane for 24 h to remove the unreacted reagents, and then dried with N_2_. All PDMS substrates were cut into 1 × 1 cm^2^ sections, which were then cleaned with acetone, ethanol, and deionized water for 10 min each. The cleaned PDMS was then placed into an argon plasma cavity for 5 min and exposed to the open atmosphere for 10 min to promote the formation of peroxides. The resulting product was then submerged in polymer aqueous solutions and irradiated under UV at 400 W for 30 min.

### 2.7. Protein Resistance Assay

The fluorescein isothiocyanate (FITC)-labeled bovine serum albumin (BSA) was used to test the protein adsorption of the zwitterionic polymer coatings. By using pristine PDMS as a control, all samples were immersed in 2 mg/mL protein solution and incubated at 37 °C for 24 h.

### 2.8. Compatibility Assay

First, fresh human blood was washed three times with PBS buffer to form a 5% concentration solution, and then the test samples were placed in 24-well plates and injected into the diluted blood solution. After being incubated at 37 °C for 2 h, all samples were briefly washed with PBS, and the process was repeated three times. The final samples were fixed with 2.5% glutaraldehyde 4 °C for 4 h. The samples were then successively immersed in graded ethanol solutions according to their concentrations, from low to high, (25, 50, 75, and 100%, *v*/*v*) for several minutes each, which allowed the red blood cells to adhere. The morphology of the adherent erythrocytes was examined using a scanning electron microscope (FET Quant 250).

## 3. Results

**Scheme 1:**
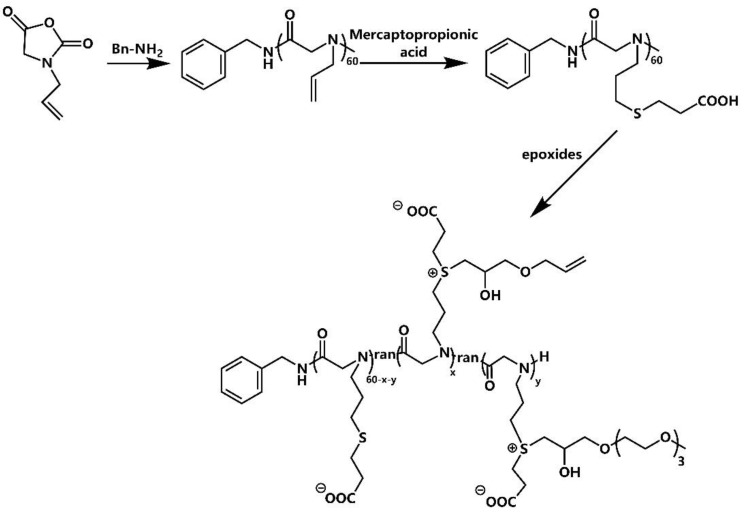
The synthetic routes of the zwitterionic polymer.

**Figure 1:**
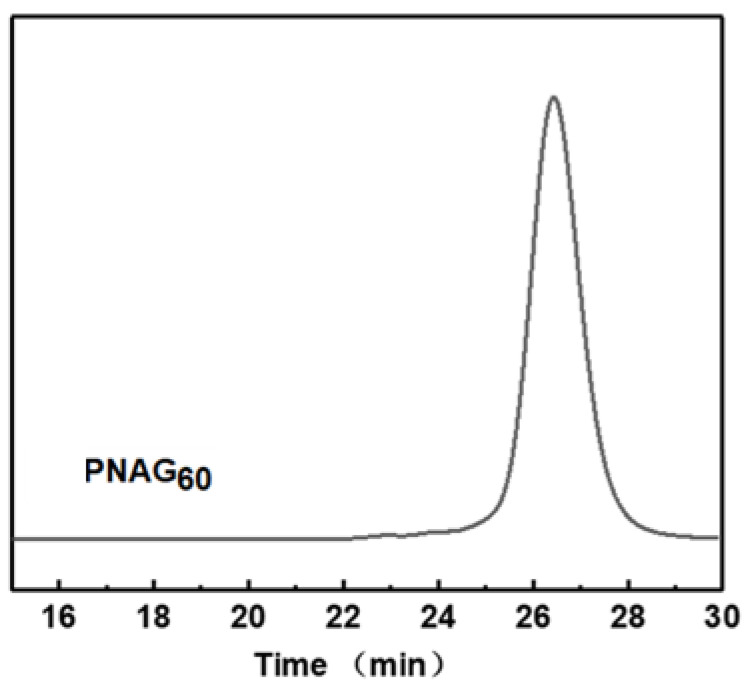
GPC chromatogram of PNAG_60_.

**Figure 2:**
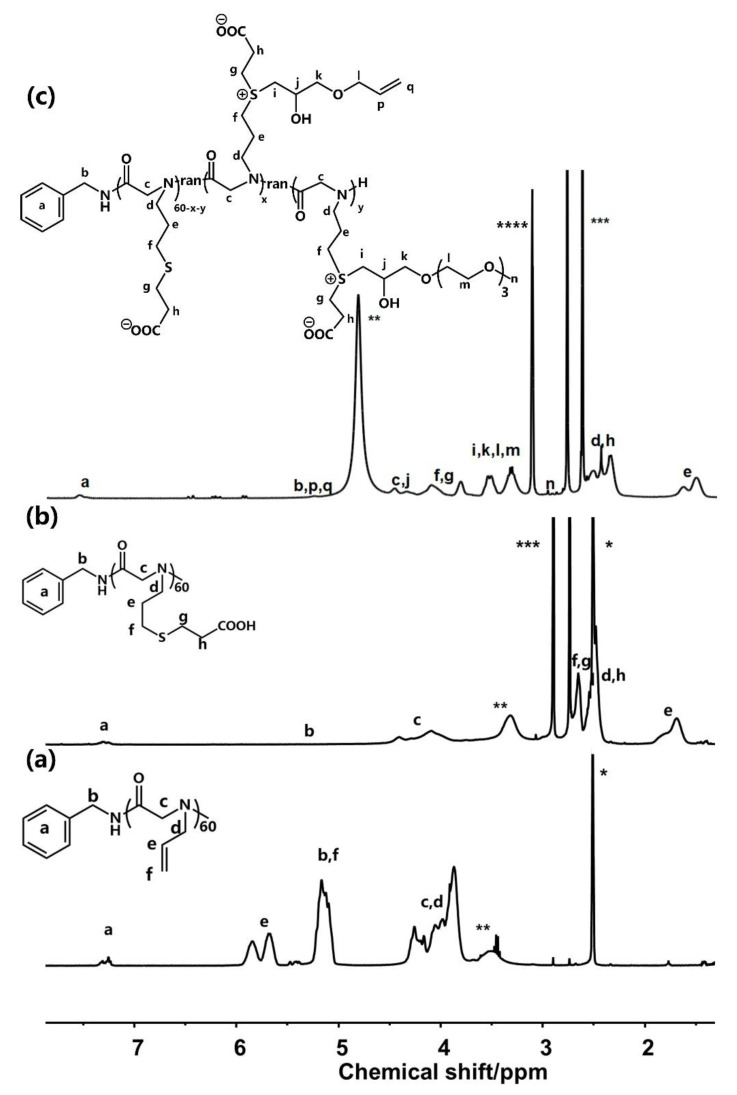
^1^H NMR spectra of the (**a**) PANG_60_ in DMSO-*d*_6_, (**b**) PNAG_60_-COOH in DMSO-*d*_6_ and (**c**) zwitterion-modified polypeptoid in CD_3_OD (* indicates DMSO-*d*_6_, ** indicates H_2_O, *** indicates DMF, and **** indicates CD_3_OD).

The synthetic route of N-allyl N-carboxyanhydride (NAG-NCA) monomers is shown in Scheme S1, while the synthetic route of the zwitterion-modified polypeptoid is shown in Scheme 1. The chemical structure of the N-allyl N-carboxyanhydride (NAG-NCA) monomers and epoxide terminated oligo (ethylene glycol) (OEG) were confirmed using ^1^H NMR spectroscopy (Figures S1 and S2). Ring-opening polymerization of *N*-substituted *N*-carboxylic anhydrides (NNCAs) with nucleophilic initiators such as primary amines has been reported to prepare the polypeptoid. Thus, ring-opening polymerization (ROP) of the NAG-NCA monomer was initiated by benzylamine. The polypeptoid with benzylamine is denoted as PNAG. The conversion of NAG-NCA monomers to a polypeptoid was shown by the two characteristic *ν*c = o peaks of the monomer when examining its FTIR spectra, while two of the characteristic carbonyl peaks at 1780–1850 cm^−1^ completely disappeared. This indicated that the NCA monomer had been completely converted into a polymer (Figure S3). The average DP (degree of polymerization) of PNAG was fixed at about 60. *n* represents the DP of the polypeptoid. The GPC trace of poly (*N*-allylglycine) showed a fairly consistent molecular weight, with a single peak of *Đ* ≤ 1.28, and narrow molecular weight distribution, which indicated that the polymerization reaction was well-controlled (Figure 1). The post-modification method of polymerization facilitated diverse polymerization reactions. When using click chemistry, the mercaptopropionic acid reacted with the double bond, causing the carboxyl group to be successfully modified on the side chain. All of the polymer’s peaks are well assigned in the ^1^H NMR spectrum, confirming its chemical structure. The nuclear magnetic ^1^H NMR is shown in Figure 2. The alkenyl proton peak at the chemical shift δ~5.01–5.98 ppm can be seen to have completely disappeared following the modification, while a new S^+^ (CH_2_)_3_ peak appeared at the chemical shift of δ~2.35–2.98 ppm, which represents the polypeptoid precursor (Figure 2b). The proton peak indicates that the total conversion of the alkenyl group reached 100%. Zwitterionic polymer was synthesized by alkylation of PNAG-COOH using two different types of functional epoxides, OEG_3_ and allyl glycidyl ether (AGE), in a given ratio of 10:1. The chemical structures and functionalization degrees of the obtained zwitterionic samples was confirmed by ^1^H NMR spectroscopy, as shown in Figure 2c. The degrees of modification of the two epoxides were determined to be 38.5% (OEG_3_) and 6.7% (AGE) from the proton integral ratios of the OEG_3_ group and allyl group to the phenyl group, respectively. The relatively low modification ratio with two epoxides was possibly due to the steric hindrance effect of the bulky substituents [35,36].

**Figure 3:**
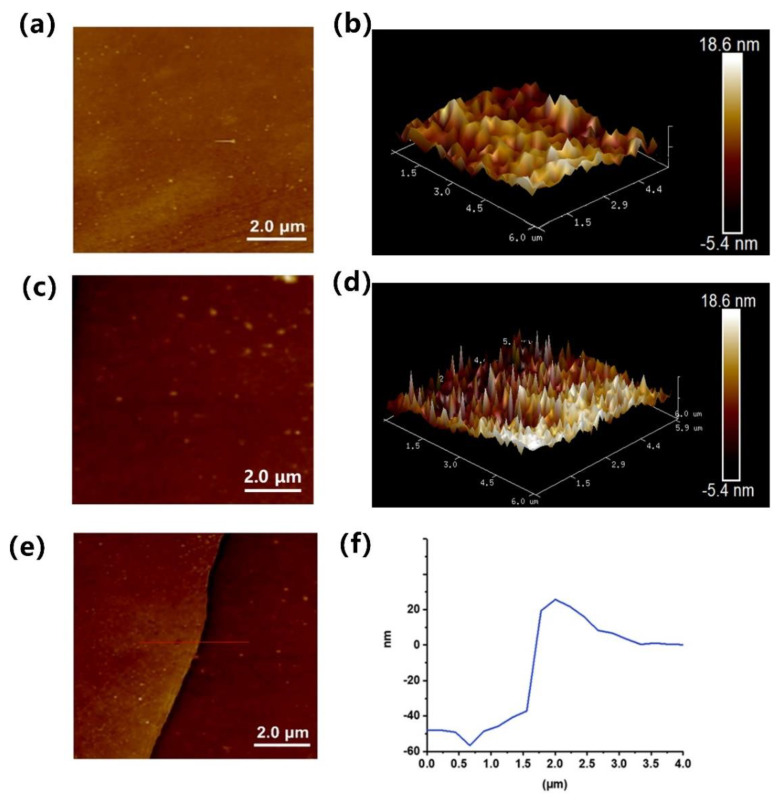
The AFM images of pristine PDMS (**a**,**b**) and zwitterionic polymer-coated PDMS (**c**,**d**). (**e**,**f**) The height profile along the line indicated in (**e**).

After a simple cleaning process, the PDMS surface was soaked in a 2 mg/mL aqueous solution of zwitterionic polymer, and the vial was placed in a UV box at room temperature for 30 min, before being gently removed with tweezers. The surface was then dried by gently blowing with nitrogen. To study the successful modification of the PDMS surface, the surface morphology was investigated. The morphologies of the smooth PDMS and PDMS with zwitterion-modified polypeptoid were observed by atomic force microscopy (AFM). As shown in Figure 3, the pristine PDMS had a relatively smooth surface. Meanwhile, when coated with a polymer, the surface roughness increased, and the polymer layer could be observed, which indicates that the zwitterion-modified polypeptoid was successfully modified on the PDMS surface. To measure the thickness of the grafted surface, half of the PDMS surface was masked to avoid plasma activation, while the other half was modified with zwitterionic polymer coating. We estimated the thickness of the coating, and from the results shown in Figure 3f, we can see that it was about 50 nm thick. The relatively thickness was possibly due to the long polymer chains and stretched conformation, which can form a hydration layer that provides effective antifouling properties [21,37,38,39]. Furthermore, static water contact angle tests were performed on PDMS before and after modification. As shown in Figure S4, pristine PDMS that was inherently hydrophobic had a water contact angle of 105.4°. In contrast, the water contact angle of zwitterionic polymer-coated PDMS fell to 70.7°, confirming the successful modification of the polymers. Note that such a grafting strategy is likely to apply to other surfaces that can generate peroxides [40].

**Figure 4:**
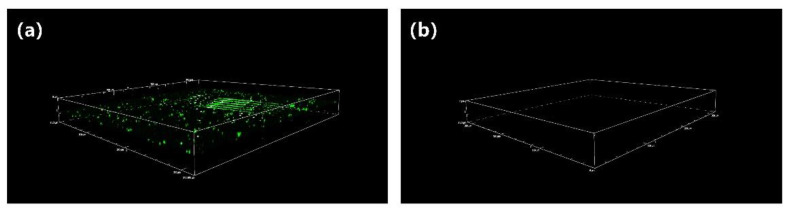
CLSM images of pristine PDMS (**a**) and zwitterionic polymer-coated PDMS (**b**).

PDMS is one of the most commonly used medical materials because of its inherent biological inertness. When PDMS is exposed to a complex environment, i.e., a foreign body, this can lead to a series of immune rejection reactions. For example, when it comes into contact with blood, proteins and red blood cells present in the blood immediately attach to the PDMS surface, followed by activation of coagulation factors, platelet adhesion, activation, and clustering, ultimately forming a so-called thrombus. As protein adsorption is the first stage of an undesired complication, biomaterials must effectively resist nonspecific protein adsorption. Thus, we used fluorescein isothiocyanate (FITC)-labeled bovine serum albumin (BSA) as a model protein to investigate the antifouling properties of smooth substrates and modified zwitterionic polymer coatings. The pristine and modified PDMS were incubated in 2 mg/mL of FITC-labeled BSA protein at 37 °C for 24 h, and then they were centrifuged at 400 rpm. The fluorescence brightness was observed via confocal laser microscopy (CLSM). Figure 4 shows many bright green fluorescent spots on the surface of the unmodified coating, indicating that a considerable amount of protein adhered to the surface of the coating. On the surface of the zwitterionic polymer coating (Figure 4b), meanwhile, barely any fluorescent spots can be seen, which indicates that next to no protein adhesion took place on the surface of the coating. The resistance of zwitterionic polymer coatings to organic adsorption stems from their super-hydrophilic properties, which allow them to attract a large number of water molecules to form a protective hydration layer, thus increasing the energy required for protein adsorption. Therefore, by inhibiting the adsorption of organic matter on its surface, we can slow down the build-up of biological pollution.

**Figure 5:**
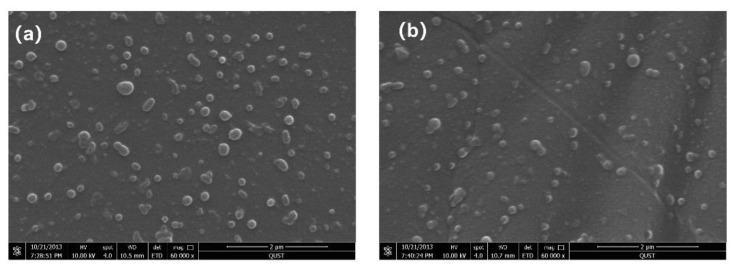
SEM images of red blood cell adhesion on (**a**) pristine PDMS and (**b**) zwitterionic polymer-coated PDMS.

Considering that zwitterionic polymer coatings show excellent antifouling properties, we further evaluated coating compatibilities using scanning electron microscopy (SEM). The morphologies of erythrocytes on PDMS surfaces with different coatings are shown in Figure 5. A certain number of red blood cells adhered to the surface of the original PDMS, and the cell membranes of the erythrocytes were intact and smooth, with no obvious traces of content flow observed (Figure 5a). Figure 5b depicts how we also discovered a similar experimental phenomenon with original PDMS, whereby the integrity of the red blood cells was preserved on the surface of PDMS. The results of SEM confirm that PDMS erythrocytes modified with zwitterionic polymers show excellent compatibility with neglected toxic side effects.

## 4. Conclusions

In summary, we successfully synthesized zwitterionic polymers with narrow molecular weight distributions using benzylamine-initiated ROP of NAG-NCA and a two-step post-modification strategy. The polymer was grafted onto the PDMS surface conveniently and quickly by using plasma and photo-initiated methods. The zwitterionic coating inhibited non-specific protein adsorption and had excellent compatibility with mammalian red blood cells. These results imply that zwitterionic polymer coatings have great potential for application in future biomedical materials and devices.

## Data Availability

The data that support the findings of this study are available in the Supplementary Material of this article.

## Supplementary Materials

The following supporting information can be downloaded at: https://www.mdpi.com/article/10.3390/biomimetics7020050/s1, Scheme S1: Synthetic route of NAG-NCA monomer; Figure S1: ^1^H NMR spectrum of N-allyl N-carboxyanhydride (NAG-NCA) monomer; Figure S2: ^1^H NMR spectrum of epoxide terminated oligo(ethylene glycol); Figure S3: FTIR spectra of NCA and polymers; Figure S4: Static water contact angle test (a) pristine PDMS and (b) zwitterionic polymer-coated PDMS.
