# Supplementary material for: Preparation of a Novel Type of Zwitterionic Polymer and the Antifouling PDMS Coating
Source: Biomimetics (Basel). 2022 Apr 21;7(2):50. doi: 10.3390/biomimetics7020050 (PMC9149847; doi:10.3390/biomimetics7020050)
Supplement: Supplementary file 1 [file biomimetics-07-00050-s001.zip › biomimetics-1652355-supplementary.pdf]

Preparation of a Novel Type of Zwitterionic Polymer and the Antifouling PDMS Coating

Xutao Ma [1], Xiaohui Fu [1] and Jing Sun [2,*]

[1] College of Polymer Science and Engineering, Qingdao University of Science and Technology, Qingdao 266042, China; 15552266138@163.com (X.M.); fuxiaohui@iccas.ac.cn (X.F.)

[2] State Key Laboratory of Supramolecular Structure and Materials College of Chemistry Jilin University, Changchun 130012, China)

* Correspondence: jingsun@jlu.edu.cn

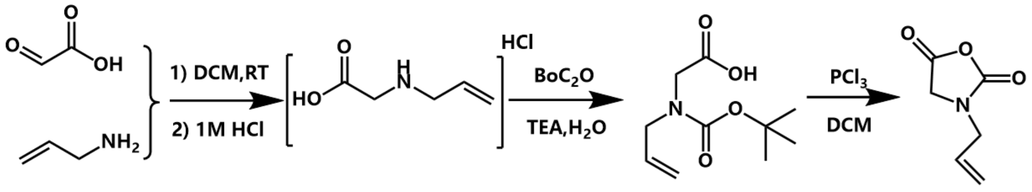

Scheme S1. Synthetic route of NAG-NCA monomer.

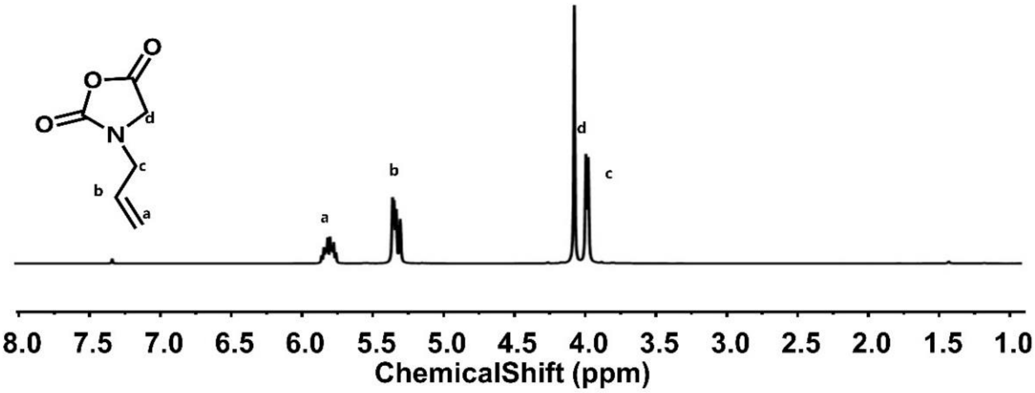

Figure S1. ^{1}H NMR spectrum of N-allyl N-carboxyanhydride (NAG-NCA) monomer.

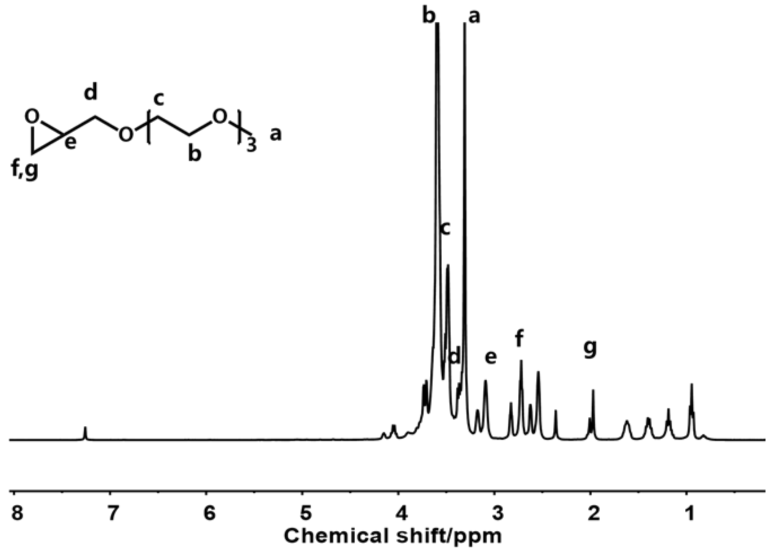

Figure S2. ^{1}H NMR spectrum of epoxide terminated oligo(ethylene glycol).

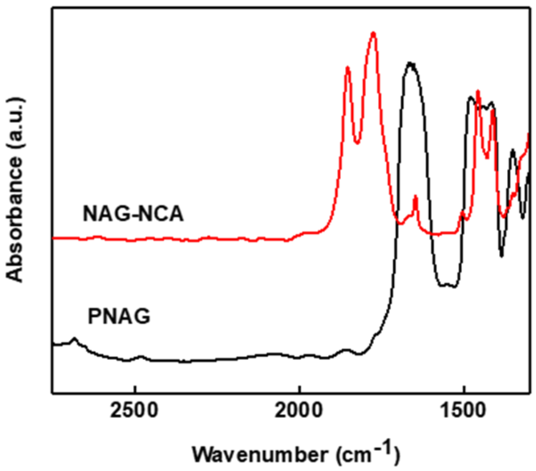

Figure S3. FTIR spectra of the NCA and polymers.

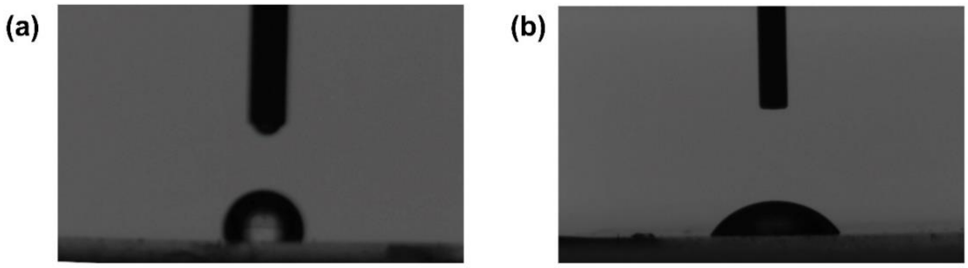

Figure S4. Static water contact angle tests of (a) pristine PDMS and (b) zwitterionic polymer-coated PDMS.
